# Combination of cyclin-dependent kinase 4/6 inhibitors and renin-angiotensin system inhibitors in breast cancer treatment: a promising therapeutic approach

**DOI:** 10.1590/1806-9282.20251164

**Published:** 2025-12-15

**Authors:** Emel Mutlu, Mevlüde İnanç

**Affiliations:** 1 Erciyes University, Medical School, Department of Medical Oncology – Kayseri, Türkiye.

**Keywords:** Breast cancer, Cyclin-dependent kinases, Renin-angiotensin system, Survival

## Abstract

**OBJECTIVE:**

The prognosis of metastatic breast cancer has improved with new targeted therapies like cyclin-dependent kinase 4/6 inhibitors. Renin-angiotensin system inhibitors, such as direct renin inhibitors, angiotensin II receptor blockers and angiotensin-converting enzyme inhibitors are frequently used to manage hypertension and heart failure. However, recent research indicates that they may also provide potential benefits in cancer therapy. The combination of renin-angiotensin system inhibitors with cyclin-dependent kinase 4/6 inhibitors is an emerging area of interest in cancer research. Our hypotesis that, by inhibiting angiogenesis, renin-angiotensin system inhibitors may reduce tumor growth and improve the effectiveness of cyclin-dependent kinase 4/6 inhibitors by cutting off the supply of nutrients and oxygen to the tumor.

**METHODS:**

Metastatic breast cancer patients treated with cyclin-dependent kinase 4/6 inhibitors were enrolled and retrospectively divided according to angiotensin-converting enzyme inhibitor/angiotensin II receptor blocker use: angiotensin-converting enzyme inhibitor/angiotensin II receptor blocker (+) and angiotensin-converting enzyme inhibitor/angiotensin II receptor blocker (-). Progression-free survival and overall survival were defined as primary and secondary endpoints. The effect of categorized data on progression-free survival and overall survival was determined by Cox regression analyses.

**RESULTS:**

Estimated median progression-free survival (HR 0.61, 95%CI 0.34–0.94; p = 0.041) and overall survival (HR 0.71, 95%CI 0.49-0.79; p: 0.01) were statistically significantly longer in the angiotensin-converting enzyme inhibitor/angiotensin II receptor blocker (+) group than in the angiotensin-converting enzyme inhibitor/angiotensin II receptor blocker (-) group.

**CONCLUSION:**

Concomitant use of renin-angiotensin system inhibitors and cyclin-dependent kinase 4/6 inhibitors may have beneficial oncological effects in patients with breast cancer beyond their cardiovascular indications.

## INTRODUCTION

Renin-angiotensin system inhibitors (RASIs) are vital for cardiovascular health. RASIs, such as direct renin inhibitors, angiotensin II receptor blockers (ARBs), and angiotensin-converting enzyme inhibitors (ACE-Is) are frequently used to manage hypertension and heart failure. However, recent research indicates that they may also provide potential benefits in cancer therapy^1^. Researchers have found that tumor-associated abnormal fibroblasts, extracellular matrix, and immune cells negatively affect the tumor microenvironment, which not only accelerates tumor progression but also reduces the effectiveness of various anticancer therapies. Beyond its effects on cardiovascular health, the RAS pathway is also implicated in tumor progression, angiogenesis, and metastasis^2,3^. Several retrospective studies suggest that patients with cancers who are using ACE-Is or ARBs may experience improved cancer-specific survival, though the exact mechanisms remain unclear^4-6^.

Survival is quite good for patients with early-stage breast cancer, and mammography screening is crucial for early diagnosis. A study conducted in Brazil reiterated the importance of mammography screening in breast cancer diagnosis^7^.

The prognosis of metastatic breast cancer has improved with new targeted therapies like cyclin-dependent kinase 4/6 (CDK 4/6) inhibitors. CDK 4/6 inhibitors, including ribociclib, palbociclib, and abemaciclib, are designed to inhibit the activity of CDK 4/6, which are critical proteins involved in regulating the cell cycle. They are especially effective in hormone receptor-positive (HR+) breast cancer, although resistance to CDK 4/6 inhibitors may develop over time^8^.

The combination of RASIs with CDK 4/6 inhibitors is an emerging area of interest in cancer research. Our hypothesis that, by inhibiting angiogenesis, RASIs may reduce tumor growth and improve the effectiveness of CDK 4/6 inhibitors by cutting off the supply of nutrients and oxygen to the tumor. By inhibiting RAS signaling, the tumor microenvironment can be modified to make it less conducive to cancer growth and more responsive to CDK 4/6 inhibitors. This could lead to a better therapeutic outcome and potential for reduced metastatic spread.

## METHODS

This study included 200 patients diagnosed with metastatic HR (+) breast cancer at Erciyes University Medical Oncology Clinic between 2019 and 2024, who received at least 3 months of CDK 4/6 inhibitor therapy during the metastatic stage. Patients who were additionally using RASIs due to hypertension comorbidity were included in the study. Patients using RASI due to comorbidities such as chronic renal failure other than hypertension were not included in the study. RASIs doses are standard doses used in hypertension according to the active substance.

Descriptive statistics and statistical analyses of the study variables were performed using SPSS 27.0 software. All tests were conducted with a 95%CI, and p<0.05 was considered statistically significant. The log-rank test and Kaplan-Meier curves were used to analyze differences in progression free survival (PFS) and overall survival (OS) between the categorized groups. Cox regression was used for the multivariate analysis on variables that showed statistical significance. To assess subgroup differences, interaction terms between treatment and concomitant medication variables were added to the Cox regression model. The significance of interaction terms was assessed using the Wald test and reported as the p-value for the interaction. The results of the analysis were expressed as medians (minimum to maximum), means, standard deviations, and hazard ratios (HR).

## RESULTS

Patients were divided into two groups according to ACE-I/ARB use: ACE-I/ARB(+) and ACE-I/ARB(-). The ACE-I/ARB(+) and ACE-I/ARB(-) groups comprised 98 (49%) and 102 (51%) patients, respectively. The mean age in the ACE-I/ARB(+) and ACE-I/ARB(-) groups was 54.5±12.5 and 50.9±10.7, respectively. There was no statistically significant difference between the groups in terms of age (p: 0.12). A total of 121 patients (60.5%) were postmenopausal. The most common histopathologic subtype was invasive ductal carcinoma (n=184, 92%). Notably, 121 (60.5%) patients were using ribociclib and 79 (39.5%) patients were using palbociclib. Also, 34 (17%) patients used thiazide diuretics and 22 (11%) patients used CCB. The partial response rate, use of thiazide diuretics, and CCB were significantly higher in the ACE-I/ARB(+) group than in the ACE-I/ARB(-) group (p<0.001, p: 0.031, p<0.001). There was no statistical difference between the ACE-I/ARB(+) and ACE-I/ARB(-) groups in terms of endocrine treatment type (aromatase inhibitor vs. fulvestrant) and CDK 4/6 inhibitor type (rabociclib vs. palbociclib) (p: 0.321, p: 0.142).

The Cox regression analysis was applied for univariate-multivariate analyses, which yielded the HR of PFS and OS related variables such as age, ACE-I/ARB use (yes vs. no), tiazide diuretics use (yes vs. no), CCB use (yes vs. no), endocrine therapy type, and CDK 4/6 type. The univariate analysis results for the association of PFS with ribociclib, aromatase inhibitors, CDK 4/6 Inhibitor treatment line ≥3, tiazide diuetics, CCB, and ACE-I/ARB use were 1.52 (95%CI 0.92–2.53; p: 0.100), 0.34 (95%CI 0.21–0.55; p<0.001), 2.25 (95%CI 1.35–3.74; p: 0.002), 0.58 (95%CI 0.26–1.28; p: 0.182), 0.56 (95%CI 0.25–1.22; p: 0.143), and 0.42 (95%CI 0.25–0.69; p: 0.001), respectively. In the multivariate analysis, aromatase inhibitors use (HR 0.42, 95%CI 0.27–0.63; p<0.001) and ACE-I/ARB use (HR 0.61, 95%CI 0.34–0.94; p: 0.041) were associated with improved PFS (Table 1). The estimated median PFS was 29.9 months for patients in the ACE-I/ARB(+) group, whereas it was 21.9 months for those in the ACE-I/ARB(-) group. This difference was found to be statistically significant (HR 0.61, 95%CI 0.34–0.94; p=0.041). To elucidate the effect of the two drugs (CCB and tiazide diuretics), we divided the patients into four subgroups for each drug according to their treatment regimen. Groups for combined use of ACE-I/ARB and CCB; ACE-I/ARB(-)#CCB(-), ACE-I/ARB(-)#CCB(+), ACE-I/ARB(+)#CCB(+), ACE-I/ARB(+)#CCB(-). In the multivariate analysis, PFS was significantly higher ACE-I/ARB(+)#CCB(-) group than ACE-I/ARB(-)#CCB(-) group (HR 0.63, 95%CI 0.31–0.77; p: 0.026). In interaction tests for subgroup analyses, no significant interaction was observed between ACE-I/ARB and CCB use (p for interaction=0.18). Groups for combined use of ACE-I/ARBand tiazide diuretics; ACE-I/ARB(-)#Tiazide(-), ACE-I/ARB(+)#Tiazide(+), ACE-I/ARB(+)#Tiazide(-), ACE-I/ARB(-)#Tiazide(+). No statistically significant difference in PFS was observed between the groups.

**Table 1:** Cox proportional hazards model for evaluating the effect of the clinical variables on progression free survival.

Variables	Categories	Median PFS (months)	Univariate analysis HR 95%Cl	p-value	Multivariate analysis HR 95%Cl	p-value
Age	≤65*	25.5				
	>65	30.2	2.16 (0.98–4.72)	0.532		
CDK 4/6 inh.	Palbociclib*	27.4				
	Ribociclib	25.2	1.52 (0.92–2.53)	0.100		
Endocrine therapy	Fulvestrant*	19.5				
	Aromatase inhibitors	29.8	0.34 (0.21–0.55)	**<0.001**	0.42 (0.27–0.63)	**<0.001**
CDK 4/6 Inh. treatment line	1-2*	28.0				
	≥3	20.7	2.25 (1.35–3.74)	**0.002**	1.42 (0.91–3.35)	0.082
Tiazide diuretics	No*	25.9				
	Yes	28.1	0.58 (0.26–1.28)	0.182		
CCB	No*	25.8				
	Yes	28.5	0.56 (0.25–1.22)	0.143		
ACE-I/ARB	No*	21.9				
	Yes	29.9	0.42 (0.25–0.69)	**0.001**	0.61 (0.34–0.94)	**0.041**
Group	ACE-I/ARB(-)#CCB(-)*	22.1				
	ACE-I/ARB(-)#CCB(+)	20	0.62 (0.14–2.59)	0.514		
	ACE-I/ARB(+)#CCB(+)	28.6	0.34 (0.10–1.12)	0.078		
	ACE-I/ARB(+)#CCB(-)	29.1	0.49 (0.29–0.82)	**0.007**	0.63 (0.31–0.77)	**0.026**
Groups	ACE-I/ARB(-)#Tiazide(-)*	22.5				
	ACE-I/ARB(+)#Tiazide(+)	28.2	0.42 (0.19–0.95)	**0.039**	0.21 (0.01–2.53)	0.321
	ACE-I/ARB(+)#Tiazide(-)	29.2	0.48 (0.28–0.83)	**0.009**	0.39 (0.08–2.63)	0.252
	ACE-I/ARB(-)#Tiazide(+)	14.6	1.7 (0.40–7.08)	0.461		

Statistically significant values are denoted in bold. CCB: calcium channel blocker; CDK 4/6: cyclin-dependent kinase 4/6; ACE-I: angiotensin-converting enzyme inhibitors; ARB: angiotensin II receptor blockers; PFS: progression free survival; HR: hazard ratio; CI: confidence interval.

* Reference category.

**Figure 1:**
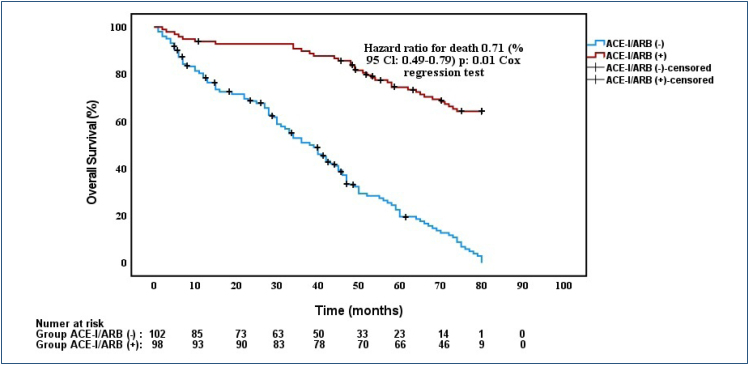
Kaplan Meier test of overall survival according to angiotensin-converting enzyme inhibitor/angiotensin II receptor blocker use.

The Cox regression analysis revealed estimated median OS of patients in the ACE-I/ARB(+) and ACE-I/ARB(-) groups was 72.2 and 66.5 months, respectively, and this was a statistically significant relationship (HR 0.71, 95%CI 0.49–0.79; p: 0.01) (Figure 1). The univariate analysis results for the association of OS with ACE-I/ARB use ACE-I/ARB(+)#CCB(-) use and ACE-I/ARB(+)#Tiazide(-) use were 0.41 (95%CI 0.20–0.86; p: 0.019), 0.42 (95%CI 0.19–0.9; p: 0.027), 0.44 (95%CI 0.20–0.97; p: 0.044). In the multivariate analysis, ACE-I/ARB use (HR 0.71, 95%CI 0.49–0.79; p: 0.01) were associated with OS. OS was significantly higher ACE-I/ARB(+)#CCB(-) group than ACE-I/ARB(-)#CCB(-) group (HR 0.79, 95%CI 0.42–0.82; p: 0.02) and ACE-I/ARB(+)#Tiazide(-) group than ACE-I/ARB(-)#Tiazide(-) group (HR 0.83, 95%CI 0.38–0.76; p: 0.04) at the Cox regression analysis (Table 2). In interaction tests for subgroup analyses, no significant interaction was observed between ACE-I/ARB and CCB use (p for interaction=0.24); additionally, no significant interaction was observed between ACE-I/ARB and thiazide use (p for interaction=0.21).

**Table 2:** Cox proportional hazards model for evaluating the effect of the clinical variables on overall survival.

Variables	Categories	Median OS (months)	Univariate analysis HR 95%Cl	p-value	Multivariate analysis HR 95%Cl	p-value
Age	≤65*	64.1				
	>65	48.2	0.89 (0.31–2.58)	0.840		
CDK 4/6 type	Ribociclib*	79.2				
	Palbociclib	72.6	1.13 (0.38–1.80)	0.639		
Endocrine therapy	Fulvestrant*	70.9				
	Aromatase inhibitors	76.9	0.59 (0.29–1.21)	0.155		
CDK 4/6 Inh. treatment line	1-2*	79.5				
	≥3	70.1	1.18 (0.54–2.61)	0.141		
Tiazide diuretics	No*	76.7				
	Yes	77.1	0.55 (0.13–2.33)	0.422		
CCB	No*	68.3				
	Yes	78.1	0.65 (0.29–2.49)	0.127		
ACE-I/ARB	No*	66.5				
	Yes	72.2	0.41 (0.20–0.86)	**0.019**	0.71 (0.49–0.79)	**0.01**
Group	ACE-I/ARB(-)#CCB(-)*	53.1				
	ACE-I/ARB(-)#CCB(+)	45.0	1.41 (0.26–4.83)	0.879		
	ACE-I/ARB(+)#CCB(+)	40	1.32 (0.36–3.50)	0.457		
	ACE-I/ARB(+)#CCB(-)	79.3	0.42 (0.19–0.90)	**0.027**	0.79 (0.42–0.82)	**0.02**
Groups	ACE-I/ARB(-)#Tiazide(-)*	67.1				
	ACE-I/ARB(+)#Tiazide(+)	77.2	0.38 (0.09–1.67)	0.200		
	ACE-I/ARB(+)#Tiazide(-)	78.4	0.44 (0.20–0.97)	**0.044**	0.83 (0.38–0.76)	**0.04**
	ACE-I/ARB(-)#Tiazide(+)	18.6	4.43 (0.56–14.9)	0.158		

Statistically significant values are denoted in bold. CCB: calcium channel blocker; CDK 4/6: cyclin-dependent kinase 4/6; ACE-I: angiotensin-converting enzyme inhibitors; ARB: angiotensin II receptor blockers; OS: overall survival; CI: confidence interval.

* Reference category.

## DISCUSSION

This retrospective study evaluated the effect of RASIs on clinical outcomes in HR (+) metastatic breast cancer patients treated with CDK 4/6 inhibitors. Our findings indicate that RASIs use is independently linked to significant improvements in both PFS and OS, implying a possible synergistic interaction between RASIs and CDK 4/6-targeted treatments.

Some preclinical studies also suggest that RASIs may enhance the efficacy of chemotherapy and targeted therapies through immune modulation and antifibrotic actions^9^. These effects may be particularly relevant in the context of CDK 4/6 inhibitors, which require effective drug penetration and a responsive tumor microenvironment to exert maximal benefit.

The angiotensin II receptor has been shown to promote tumor growth, angiogenesis, inflammation, fibrosis, and metastatic progression. By inhibiting this pathway, RASIs may suppress key protumorigenic processes^10^. In a previous study, 168 patients diagnosed with early stage II/III breast cancer who used ACEI or ARB for at least 6 months and 15% of the ACEI/ARB users experienced recurrence, compared to 23% in nonusers. Additionally, the disease-free survival of ACEI/ARB users was significantly higher than that of nonusers, suggesting that the use of ACEI/ARB was linked to a reduced risk of breast cancer recurrence^11^. In a study conducted on patients with hepatocellular carcinoma (HCC), it was observed that the use of ARBs after radiofrequency ablation reduced the recurrence rate^12^. A similar benefit has been observed not only in breast cancer and HCC but also in the recurrence of colorectal cancer (CRC), particularly in left-sided CRC and early-stage CRC^13^.

Our study, the median PFS was significantly longer in patients using ACE-I/ARB (29.9 months) compared to those who did not use them (21.9 months), with ACE-I/ARB use remaining a statistically significant factor in the multivariate model (HR 0.61, 95%CI 0.34–0.94; p=0.041). Likewise, a survival benefit was observed in terms of OS (72.2 vs. 66.5 months; HR 0.71, 95%CI 0.49–0.79; p: 0.01), suggesting that RASIs may contribute to prolonged disease control and potentially delay cancer-related mortality in this population. Although the 5.7-month difference in OS seems numerically modest, this period is very important for survival in cancer patients. The RAS expression could not be studied at the cellular level from tumor tissues because we did not have sufficient technical means. We hope that if we could perform the expression analysis, we could obtain stronger results in our study.

A notable finding in our study was that the survival benefit of ACE-I/ARB was more pronounced in patients not using concurrent calcium CCBs or thiazide diuretics. Specifically, the ACE-I/ARB(+)#CCB(-) subgroup demonstrated significantly longer PFS and OS compared to the ACE-I/ARB(-)#CCB(-) group, while the ACE-I/ARB(+)#CCB(+) group did not show the same magnitude of benefit. Similarly, among patients not receiving thiazide diuretics, ACE-I/ARB use remained associated with superior OS, whereas no significant PFS differences were observed between tiazide subgroups. This may be due to the small number of patients in our subgroup. Our study could be a pioneer for conducting different studies with larger samples. These results may point to a pharmacodynamic interaction between antihypertensive classes, or differential effects on tumor biology. For instance, certain CCBs, particularly nondihydropyridines, have been associated with altered drug metabolism via cytochrome P450 inhibition, which could influence the pharmacokinetics of CDK 4/6 inhibitors. Alternatively, CCBs and thiazides may lack the tumor-suppressive effects observed with RAAS inhibitors or may exert counter-regulatory effects on pathways relevant to tumor progression. While speculative, these findings merit further exploration in future pharmacologic and translational studies.

Consistent with previous studies, our analysis also showed that the aromatase inhibitor use was independently associated with improved PFS, likely reflecting their established efficacy in HR(+) breast cancer. However, different resistance mechanisms prompt the search for new treatments^14^.

A previous study found that estrogen replacement therapy, used to reduce menopausal symptoms in postmenopausal patients with breast cancer, increased the risk of breast cancer, and it was suggested that the concurrent use of melatonin may reduce this risk. This suggests that the effectiveness of drugs used for breast cancer may be altered by interactions with other drugs at the cellular level^15^.

When we look at cost-effectiveness, cancer drugs are quite expensive, and unfortunately, treatment options are limited. RASIs are considerably cheaper than cancer drugs, and considering their contribution to survival, their use in cancer patients can be considered cost-effective.

However, the retrospective design imposes certain limitations, including susceptibility to selection bias, unmeasured confounding, and incomplete data on medication adherence, dose intensity, and RAS component expression in tumor tissues. The relatively small sample size in certain subgroups (e.g., CCB and thiazide users) also limits the power to detect subtle differences or interactions. Furthermore, while the associations are compelling, causality cannot be established without randomized prospective studies.

These findings are of clinical importance, particularly considering the high prevalence of hypertension and cardiovascular comorbidities in breast cancer patients, especially in postmenopausal women and those undergoing endocrine therapy. The dual benefit of cardiovascular risk management and potential oncologic advantage positions ACE-I/ARB as a preferred class of antihypertensives in this setting. Given the widespread use of ACE-Is and ARBs, even a modest survival benefit could have meaningful population-level impact. Future prospective trials and mechanistic studies are needed to validate these results, explore the optimal timing and duration of RAS blockade, and examine whether certain patient subgroups derive greater benefit based on tumor biology or genetic factors.

Additionally, integrating biomarker studies to assess angiotensin receptor expression, tumor microenvironment remodeling, and immune infiltration may help elucidate the mechanisms by which RASIs influence breast cancer progression and treatment response.

## CONCLUSION

Our study shows that the use of RASIs is independently linked to improved PFS and OS in patients with HR (+) metastatic breast cancer undergoing treatment with CDK 4/6 inhibitors. These findings suggest that RASIs may have beneficial oncologic effects beyond its cardiovascular indications. Prospective clinical trials are warranted to confirm these observations and to define the optimal use of RAS targeting agents in the management of breast cancer.

## Data Availability

The datasets generated and/or analyzed during the current study are available from the corresponding author upon reasonable request.

## References

[1] Haliga RE, Cojocaru E, Sîrbu O, Hrițcu I, Alexa RE, Haliga IB (2025). Immunomodulatory effects of RAAS inhibitors: beyond hypertension and heart failure. Biomedicines.

[2] Bryce AS, Dreyer SB, Froeling FEM, Chang DK (2022). Exploring the biology of cancer-associated fibroblasts in pancreatic cancer. Cancers (Basel).

[3] Yang J, Yang X, Gao L, Zhang J, Yi C, Huang Y (2021). The role of the renin-angiotensin system inhibitors in malignancy: a review. Am J Cancer Res.

[4] Tang H, Abston E, Sojoodi M, Wang Y, Erstad DJ, Lin Z (2024). An angiotensin system inhibitor (losartan) potentiates antitumor efficacy of cisplatin in a murine model of non-small cell lung cancer. JTCVS Open.

[5] Sun H, Li T, Zhuang R, Cai W, Zheng Y (2017). Do renin-angiotensin system inhibitors influence the recurrence, metastasis, and survival in cancer patients?: evidence from a meta-analysis including 55 studies. Medicine (Baltimore).

[6] Keith SW, Maio V, Arafat HA, Alcusky M, Karagiannis T, Rabinowitz C (2022). Angiotensin blockade therapy and survival in pancreatic cancer: a population study. BMC Cancer.

[7] Rivas FWS, Gonçalves R, Mota BS, Sorpreso ICE, Toporcov TN, Filassi JR (2024). Comprehensive diagnosis of advanced-stage breast cancer: exploring detection methods, molecular subtypes, and demographic influences - a cross-sectional study. Clinics (Sao Paulo).

[8] Yousuf M, Alam M, Shamsi A, Khan P, Hasan GM, Rizwanul Haque QM (2022). Structure-guided design and development of cyclin-dependent kinase 4/6 inhibitors: a review on therapeutic implications. Int J Biol Macromol.

[9] Vallejo-Ardila DL, Fifis T, Burrell LM, Walsh K, Christophi C (2018). Renin-angiotensin inhibitors reprogram tumor immune microenvironment: a comprehensive view of the influences on anti-tumor immunity. Oncotarget.

[10] Afsar B, Afsar RE, Ertuglu LA, Kuwabara M, Ortiz A, Covic A (2021). Renin-angiotensin system and cancer: epidemiology, cell signaling, genetics and epigenetics. Clin Transl Oncol.

[11] Chae YK, Valsecchi ME, Kim J, Bianchi AL, Khemasuwan D, Desai A (2011). Reduced risk of breast cancer recurrence in patients using ACE inhibitors, ARBs, and/or statins. Cancer Invest.

[12] Facciorusso A, Prete V, Crucinio N, Muscatiello N, Carr BI, Leo A (2015). Angiotensin receptor blockers improve survival outcomes after radiofrequency ablation in hepatocarcinoma patients. J Gastroenterol Hepatol.

[13] Zeman M, Skałba W, Wilk AM, Cortez AJ, Maciejewski A, Czarniecka A (2022). Impact of renin-angiotensin system inhibitors on the survival of patients with rectal cancer. BMC Cancer.

[14] Will M, Liang J, Metcalfe C, Chandarlapaty S (2023). Therapeutic resistance to anti-oestrogen therapy in breast cancer. Nat Rev Cancer.

[15] Soares JM, Mota BS, Nobrega GB, Filassi JR, Sorpreso ICE, Baracat EC (2023). Breast cancer survivals and hormone therapy: estrogen and melatonin. Rev Assoc Med Bras (1992).

